# Microbiota-derived tryptophan metabolites indole-3-lactic acid is associated with intestinal ischemia/reperfusion injury via positive regulation of YAP and Nrf2

**DOI:** 10.1186/s12967-023-04109-3

**Published:** 2023-04-18

**Authors:** Fang-Ling Zhang, Xiao-Wei Chen, Yi-Fan Wang, Zhen Hu, Wen-Juan Zhang, Bo-Wei Zhou, Peng-Fei Ci, Ke-Xuan Liu

**Affiliations:** 1 Department of Anesthesiology, Nanfang Hospital, Southern Medical University, 1838 Guangzhou Ave N, Guangzhou, 510515 China; 2 Department of Anaesthesiology, The University of Hong Kong-Shenzhen Hospital, Shenzhen, 518053 Guangdong China

**Keywords:** Intestinal ischemia/reperfusion injury, Indole-3-lactic acid, Oxidative stress, YAP, Nrf2

## Abstract

**Background:**

*Lactobacillus* has been demonstrated to serve a protective role in intestinal injury. However, the relationship between *Lactobacillus murinus* (*L. murinus*)-derived tryptophan metabolites and intestinal ischemia/reperfusion (I/R) injury yet to be investigated. This study aimed to evaluate the role of *L. murinus*-derived tryptophan metabolites in intestinal I/R injury and the underlying molecular mechanism.

**Methods:**

Liquid chromatograph mass spectrometry analysis was used to measure the fecal content of tryptophan metabolites in mice undergoing intestinal I/R injury and in patients undergoing cardiopulmonary bypass (CPB) surgery. Immunofluorescence, quantitative RT-PCR, Western blot, and ELISA were performed to explore the inflammation protective mechanism of tryptophan metabolites in WT and Nrf2-deficient mice undergoing intestinal I/R, hypoxia-reoxygenation (H/R) induced intestinal organoids.

**Results:**

By comparing the fecal contents of three *L. murinus*-derived tryptophan metabolites in mice undergoing intestinal I/R injury and in patients undergoing cardiopulmonary bypass (CPB) surgery. We found that the high abundance of indole-3-lactic acid (ILA) in the preoperative feces was associated with better postoperative intestinal function, as evidenced by the correlation of fecal metabolites with postoperative gastrointestinal function, serum I-FABP and D-Lactate levels. Furthermore, ILA administration improved epithelial cell damage, accelerated the proliferation of intestinal stem cells, and alleviated the oxidative stress of epithelial cells. Mechanistically, ILA improved the expression of Yes Associated Protein (YAP) and Nuclear Factor erythroid 2-Related Factor 2 (Nrf2) after intestinal I/R. The YAP inhibitor verteporfin (VP) reversed the anti-inflammatory effect of ILA, both in vivo and in vitro. Additionally, we found that ILA failed to protect epithelial cells from oxidative stress in Nrf2 knockout mice under I/R injury.

**Conclusions:**

The content of tryptophan metabolite ILA in the preoperative feces of patients is negatively correlated with intestinal function damage under CPB surgery. Administration of ILA alleviates intestinal I/R injury via the regulation of YAP and Nrf2. This study revealed a novel therapeutic metabolite and promising candidate targets for intestinal I/R injury treatment.

**Supplementary Information:**

The online version contains supplementary material available at 10.1186/s12967-023-04109-3.

## Background

Intestinal ischemia/reperfusion (I/R) injury is a common pathophysiological process that causes high mortality rates. The etiology includes infection, trauma and hemorrhagic shock, as well as certain abdominal surgeries, such as small bowel transplantation, abdominal aortic aneurysm replacement, intestinal obstruction, and cardiopulmonary bypass (CPB) surgery, which lead to acute mesenteric ischemia [1, 2]. Intestinal I/R not only causes intestinal injury but also triggers systemic inflammatory responses that lead to morphological as well as functional changes in remote organs and life‐threatening multiple organ dysfunction syndrome (MODS) [3, 4]. Despite considerable improvements in surgical techniques and perioperative management in the last two decades, the mortality rate of MODS patients who undergo intestinal I/R injury remains high, approximately 67–80% [5, 6]. Effective approaches are urgently needed in treating intestinal I/R injury.

Although the pathogenic mechanisms involved in the progression of I/R injury are not yet fully understood, it is generally agreed that reperfusion may aggravate the degree of injury by inducing a powerful systemic inflammatory response, characterized by the production of pro-inflammatory cytokines, reactive oxygen species (ROS), inflammatory transcription factor activation, as well as other pro-inflammatory mechanisms [7]. Such events eventually lead to dysbiosis of intestinal microbiota, and bacterial translocation [8]. Significantly, accumulating evidence shows that the metabolic products secreted or regulated by the intestinal microbiome serve as potential treatment for a variety of diseases related to dysbacteriosis [9–12]. Our group has previously found that a high abundance of *Lactobacillus murinus* (*L. murinus*) in intestinal I/R-resistant mice correlated positively with gastrointestinal function under intestinal I/R injury [13]. Mono-colonization with *L. murinus* isolates exhibited fecal tryptophan metabolites including indole-3-lactic acid (ILA), indole-3-aldehyde (IAld) and indole-3-acetic acid (IAA) compared with control germ-free mice [14]. Tryptophan metabolites generated by intestinal flora play a vital role in physiological and biochemical functions [14]. Tryptophan metabolites are often used as aryl hydrocarbon receptor (AhR) ligands to regulate innate and adaptive immunity, cell proliferation, inflammation and apoptosis [12, 15, 16]. Nonetheless, the role of *L. murine*-derived tryptophan metabolites in intestinal I/R injury remains unclear.

Research has demonstrated that cytoplasmic AhR can activate ERK and YAP signaling [17, 18]. The canonical Hippo signaling pathway as well as its downstream effector Yes-associated protein (YAP) control the size of animal organs by regulating cellular proliferation, cell differentiation and apoptosis [19, 20]. Upon activating the Hippo pathway, mammalian sterile20-like kinase 1 and 2 (Mst1/2) in complex with regulatory protein SAV1, phosphorylates and activates large tumor suppressor 1 and 2 (Lats1/2) kinase in complex with regulatory protein MOB1, Lats1/2 kinase then phosphorylates and inactivates YAP/TAZ, resulting in its cytoplasmic isolation and degradation [20]. In the absence of active Lats1/2 kinases, YAP/TAZ remains active and translocates to the nucleus, where it is involved in cell proliferation and apoptosis [21]. Therefore, we hypothesized that tryptophan metabolites upregulate Yap expression through AhR receptors. Previous studies suggest that crosstalk between Nrf2 and YAP decreases chemotherapeuticsensitivity in urothelial cancer [22, 23]. Downregulation of YAP and Nrf2 protein expression reduces pancreatic cancer cell migration [24]. However, it remains unknown whether YAP and Nrf2 are involved in the mechanism of tryptophan metabolites under intestinal I/R injury.

With the aim to reveal the unknown role and potential molecular mechanisms of *L. murinus*-derived tryptophan metabolites in intestinal I/R injury, we first analyzed the contents of three *L. murinus*-derived tryptophan metabolites in mice under intestinal I/R injury and correlated patients’ fecal metabolites with postoperative gastrointestinal function in patients undergoing CPB surgery. We further examined the role of ILA in treating intestinal I/R injury and explored the hypothesis that the protective mechanisms of ILA are via the regulation of YAP and Nrf2. This study will help to elucidate a novel mechanism of intestinal I/R injury and reveal a new therapeutic strategy for clinical practice.

## Methods

### Animal studies

6–8 weeks old male C57BL/6 mice (Nanfang Hospital of Southern Medical University, Guangzhou, China) or Nrf2-deficient mice (breeding pair provided by Dr. Lei Gao, Southern Medical University) on a C57BL/6 background were kept in standard conditions and acclimatized for three days prior to the experiment. They were gavaged with either 0.1 mL/20 g of corn oil (C8267, Sigma Aldrich) or 20 mg/kg of ILA (832-97-3, Targetmol) once a day for 7 days. Prior to surgery, the mice were given an intraperitoneal injection of YAP inhibitor (verteporfin (VP), SML0534, Sigma Aldrich) at a dose of 100 mg/kg. The mice were then randomized into various groups: (1) Sham; (2) I/R; (3) I/R + ILA; (4) I/R + ILA + VP; (5) I/R + VP. The sham group who were given 0.1 mL/20 g of corn oil once a day for 7 days were modeled by intestinal I/R injury without clamping the superior mesenteric artery while the I/R group received 0.1 mL/20 g of corn oil once a day for seven days were modeled by intestinal I/R injury. The model of intestinal I/R injury used in this experiment was described previously [25]. In brief, the superior mesenteric arteries of mice were clamped for 60 min, supplemented with 0.5 mL saline before clamping and after reperfusion, and the mice were sacrificed two hours after the clip removal. During this period, the mice were placed on a heating pad to maintain the body temperature at 37 °C. Blood samples were collected immediately after the sacrifice. Serum and necrotic ileum samples were collected. The Nanfang Hospital of Southern Medical University Committee approved this study (approval number NFYY-2020-0653).

### Participants

Patients who undergo elective cardiopulmonary bypass surgery are considered to have experienced intestinal ischemia/reperfusion [26, 27]. The inclusion and exclusion criteria of enrollment were as previously described [25]. In short, the inclusion criterions were patients who provided written informed consent, aged 18 to 75 years old and required elective heart valve replacement or coronary artery bypass grafting surgery in the cardiology department of Nanfang Hospital, Southern Medical University. The key exclusion criteria were those with chronic or acute kidney disease, or diseases that may cause intestinal ischemia such as previous gastrointestinal surgery or chronic digestive system diseases and emergency surgery. The experimental design was reviewed by the Ethical Committee of Nanfang Hospital, Southern Medical University (approval number NFEC-202009-k2-01), and all patients provided written informed consent.

During a period of 6 months, patients were identified and fecal samples were collected before surgery. Blood samples were collected at pre-operative (T0) and after sternum closure (T1). The collected blood samples were centrifuged at 3000 rpm for 10 min at 4 °C, and the serum was aliquoted and frozen at − 80 °C for subsequent analysis.

### Organoid cultures

The mice intestinal organoids were extracted and cultured as described in our previous publication [25]. In this experiment, the proximal small intestinal crypts were obtained from mice aged 4–8 weeks old, and were cultured in matrigel drops (356231, Coning). IntestiCult medium (06005, STEMCELL Technologies) was used to promote cell confluence and differentiation. For hypoxia-reoxygenation (H/R) experiments, organoids were precultured in ILA (0.5 mM), CH-223191 (10 μM, T2448, TargetMol) or VP (5 μM) for 24 h. It was then placed in a 95% N_2_-5% CO_2_ humidity incubator for 12 h and further moved into a 95% O_2_-5% CO_2_ incubator for 4 h. The organoids were randomized into five groups before being subjected to H/R experiments: (1) Control + DMSO; (2) H/R + DMSO; (3) H/R + ILA; (4) H/R + ILA + VP; (5) H/R + VP. The negative control (NC) group was treated with DMSO without H/R injury while the H/R group was treated with DMSO and subjected to H/R injury.

### Lausanne intestinal failure estimation (LIFE) score

The post-operative gastrointestinal failure score of patients was evaluated by researchers who were double-blinded. This score was based on a scale of 0–4 according to the LIFE criteria [28]. The highest value was used as the intestinal score. In brief, the scoring criteria were a combination of multiple variables such as intra-abdominal pressure, enteral nutrition, hyperlactatemia, gastric residue, bowel sounds, constipation and diarrhea.

### Human FABP2/I-FABP immunoassay

Patients’ serum I-FABP levels were determined using Human FABP2/I-FABP Immunoassay kits (DFBP20, R&D Systems, Inc., USA) as instructed by the manufacturer.

### d-Lactate assay kit

Patients’ serum D-lactate levels were determined using D-Lactate Assay Kit (Colorimetric) (ab83429, Abcam, USA) as instructed by the manufacturer.

### Lactate dehydrogenase (LDH) levels

LDH levels in culture medium and serum were determined using LDH kits (A020-2-2, Nanjing Jiancheng Bioengineering Institute, China) as instructed by the manufacturer.

### Liquid chromatograph mass spectrometry

Feces collected from patients were used for targeted analysis. Targeted quantitative detection of ILA, IAA and IAld were performed using Thermo Scientific™ TSQ Quantiva™. Standard IAA (I2886, Sigma Aldrich), ILA (I89850, Shanghai Acmec Biochemical Co., Ltd) and IAld (I90651, Shanghai Acmec Biochemical Co., Ltd) were dissolved in methanol/water (v/v = 4/1) for the preparation of the standard curve. Sample preparation was done according to previously published methods with a slight modification [14]. Briefly, 50 mg of fecal sample was weighed, thawed on an ice bath and mixed with 1 ml methanol/water (v/v = 4/1). This mixture was vortexed and dissolved under sonication for 10 min. The mixture was then centrifuged at 12,000 g for 10 min at 4 °C. The supernatant was filtered through a 0.22 mm nylon filter and transferred to the sample vials. Chromatographic separation was performed using a Prelude SPLC™ sample preparation and a liquid phase system (Thermo Fisher Scientific Corp., USA). Quantitative detection was performed using Thermo Scientific™ TSQ Quantiva™. Raw data processing and peak area statistics were analyzed with TraceFinder™ software version 3.3 SP1 (Thermo Fisher Scientific Corp., USA).

### CellTiter-Glo 3D cell viability assay

The small intestinal organoids were seeded at 30 organoids per well in an opaque 96-well plate, and different concentrations of ILA were added to the experimental group. The culture or modeling steps were carried out according to the above methods. CellTier-Glo 3D reagent (G9683, Promega, USA) and culture medium were mixed in a 1:1 ratio and added to each well. The contents were immediately mixed vigorously for 10 min and incubated for 20 min at room temperature. The content was mildly mixed before detection.

### Cytokine measurements

Serum was analyzed for tumor necrosis factor α (TNFα), interleukin-6 (IL-6) and interleukin-1β (IL-1β) using the Mouse TNFα ELISA Kit (KE10002, Proteintech, China), Mouse IL-6 ELISA Kit (KE10007, Proteintech, China), Mouse IL-1β ELISA Kit (KE10003, Proteintech, China) according to the manufacturer’s instructions. Cell supernatants were assayed according to the manufacturer’s instructions and normalized to the cell count.

### Western blot

Proteins from ileum samples and organoids were extracted using RIPA lysis buffer (P0013B, Beyotime, China) and separated on a 12% SDS-PAGE gel. The proteins were then transferred onto a nitrocellulose membrane, which was blocked with 5% bovine serum albumin for 1 h, and then incubated overnight with YAP (sc-101199, Santa Cruz Biotechnology) antibody, Phospho-YAP (Ser127) (13008 T, Cell Signaling Technology, USA) antibody, Nrf2 (16396-1-AP, Proteintech, China) antibody and GAPDH antibody (10494–1-AP, Proteintech, China) at 4 °C. The membrane was then incubated with horseradish peroxidase-conjugated secondary antibody (SA00001-1, SA00001-2, Proteintech, China) for 1 h at room temperature. After the application of enhanced chemiluminescence (32209, Thermo Fisher Scientific, Inc., USA), imaging was done using the COMPLEX^TM^2000 Automated Chemiluminescent Imaging System (Bioworld, Shanghai, China).

### RNA isolation and quantitative RT-PCR

Total RNA from ileum samples and organoids was extracted using Trizol reagent (15596026, Invitrogen, USA). cDNA was transcribed using a 96-Well Thermal Cycler Veriti™ (Applied Biosystems, USA). Quantitative PCR was performed using the QuantStudio™ 6 Pro Real-Time PCR System (Applied Biosystems, USA). Primer sequences are shown in Additional file 1: Table S1. Target gene mRNA expressions were calculated and normalized to housekeeping gene 18 s mRNA level.

### Terminal Deoxynucleotidyl Transferase (Tdt)-mediated dUTP Nick End Labeling (TUNEL) staining

The TUNEL assay was performed using the In situ Fluorometric TUNEL kit (11,684,795,910, Roche, Switzerland) according to the manufacturer’s instructions. Three to six random fields from each ileum samples and organoids were captured and quantified at a 200 × magnification using the ortho fluorescence microscope (Zeiss Axio Imager D2, German). The number of positive nuclei was confirmed by fluorescence microscope and measured with ImageJ software. The ratio of positive cell counts to all cells in the villi was calculated.

### Hematoxylin and eosin (H&E) staining

Hematoxylin eosin staining was performed to observe the intestinal histological features. Ileum samples were obtained, fixed in 4% paraformaldehyde, paraffin-embedded, and sliced into 4-μm-thick sections. The intestinal histologic pathology was scored according to the Chiu scoring system and analyzed blindly [29]. Five to ten random fields were scored per animal. Images were captured at 200 × using an Olympus fluorescence microscope (Olympus, Tokyo, Japan).

### Immunofluorescence and immunohistochemistry

Organoid samples were obtained, fixed in 4% paraformaldehyde at 4℃ for 1 h, dehydrated in 15–30% sucrose for 2 days. The organoid was embedded in OCT and sliced into 8-μm-thick sections. Ileum and organoid sections were stained with primary mAbs against ZO-1 (ab96587, Abcam), Occludin (ab216327, Abcam), Ki67 (ab279653, Abcam), Lysozyme (A0099, Dako), Muc2 (27675-1-AP, Proteintech), and YAP. Images at a 200 × magnification were captured using a Zeiss Axio Imager D2 immunofluorescence microscope. Three to six random fields were quantified per section. Immunohistochemistry was performed as previously described [25]. The relative intensities of stained proteins were determined by automated image analyses in three to five randomly selected 200 × fields for every sample.

### Malondialdehyde (MDA) levels

MDA levels in serum and culture medium were determined using MDA kits (A003-4-1, Nanjing Jiancheng Bioengineering Institute, China) as instructed by the manufacturer.

### Glutathione (GSH) levels

GSH levels in serum and culture medium were determined using GSH kits (A006-2-1, Nanjing Jiancheng Bioengineering Institute, China) as instructed by the manufacturer.

### Statistical analysis

GraphPad Prism software (version 8.0) and SPSS (version 26.0) were used for data analyses by investigators blinded to group allocations. We checked the normality of data by Shapiro-Wilk normality test before analysis. Two-tailed student’s t-test, one-way ANOVA and two-way ANOVA were used when the data were normally distributed. The findings are presented as mean ± SEM, and a P-value < 0.05 indicates a statistically significant difference.

## Results

### Reduced stool indole-3-lactic acid levels correlate with post-operative intestinal dysfunction and tissue injury

**Fig. 1:**
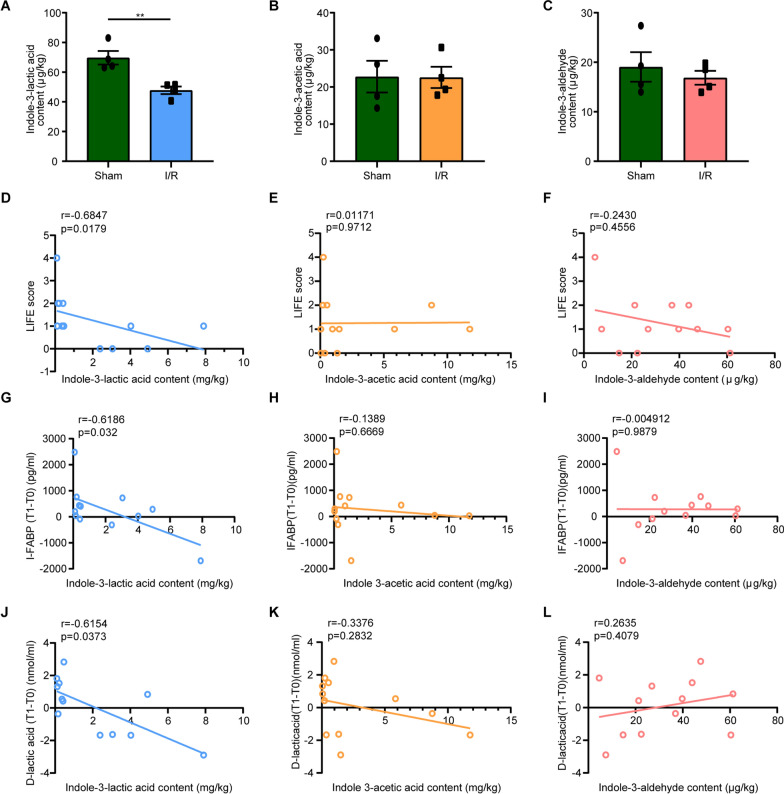
Correlation between ILA, IAA, IAld levels with intestinal I/R injury. **A**-**C** The level of ILA (**A**), IAA (**B**), IAld (**C**) in cecal contents from the sham mice and mice undergoing intestinal I/R injury (n = 4). **D**-**F** Correlation analysis between preoperatively fecal contents of ILA (**D**), IAA (**E**), IAld (**F**) levels and LIFE score in CPB surgery patients (n = 12). **G-I** Correlation analysis between preoperatively fecal contents of ILA (**G**), IAA (**H**), IAld (**I**) levels and serum I-FABP levels of patients after surgery (T1) as compared to preoperatively (T0) (n = 12). **J-L** Correlation analysis between pre-operative fecal content of ILA (**J**), IAA (**K**), IAld (**L**) levels and serum D-lactate levels of patients after surgery (T1) as compared to preoperatively (T0) (n = 12). Results were presented as mean ± SEM. The statistical tests used included: two-tailed student’s t-test in **A**–**C** and Spearman’s correlation coefficients in **D**–**L**. ** p < 0.01

To investigate the potential role of *L. murinus*-derived tryptophan metabolites in intestinal I/R injury, the cecal contents of ILA, IAA and IAld were evaluated in the sham group and the intestinal I/R group. The content of ILA was significantly lower in the I/R group compared to the sham group (Fig. 1A), whereas the content of IAA and IAld showed similar levels between the two groups (Fig. 1B, C). The CPB surgery usually lasts for several hours, which is easy to develop low blood circulation, resulting in intestinal ischemia during the operation, and develop intestinal I/R injury after the operation [30]. The LIFE score is used to evaluate patients’ intestinal function [28]. Interestingly, we discovered a significant negative correlation between fecal ILA levels and LIFE scores among patients undergoing CPB surgery (Fig. 1D). However, we did not find a significant correlation between fecal IAA and IAld level and LIFE scores (Fig. 1E, F). These findings suggest that the decline in ILA levels is associated with intestinal injury.

Serum I-FABP and D-Lactate levels were evaluated as potential biomarkers for intestinal injury [26]. Higher level of serum I-FABP and D-Lactate indicate more severe intestinal damage. We compared the correlation between fecal ILA levels and serum I-FABP and D-lactate levels before and after surgery. We found that the serum I-FABP level showed a significant negative correlation with the fecal ILA level in patients at T1 as compared to T0 (Fig. 1G). There was no significant correlation between IAA and IAld levels with serum I-FABP levels at T1 as compared to T0 (Fig. 1H, I). Similarly, we found that serum D-lactate levels had a significant negative correlation with fecal ILA level in patients at T1 as compared to T0 (Fig. 1J). Whereas no significant correlation between IAA and IAld levels with serum D-Lactate levels at T1 as compared to T0 (Fig. 1K, L). These data suggest that high abundance of ILA level is associated with better intestinal function and administration with ILA has a potential protective role in intestinal I/R injury.

### Indole-3-lactic acid protects enterocytes from death and inflammatory damage both in vivo and in vitro

**Fig. 2:**
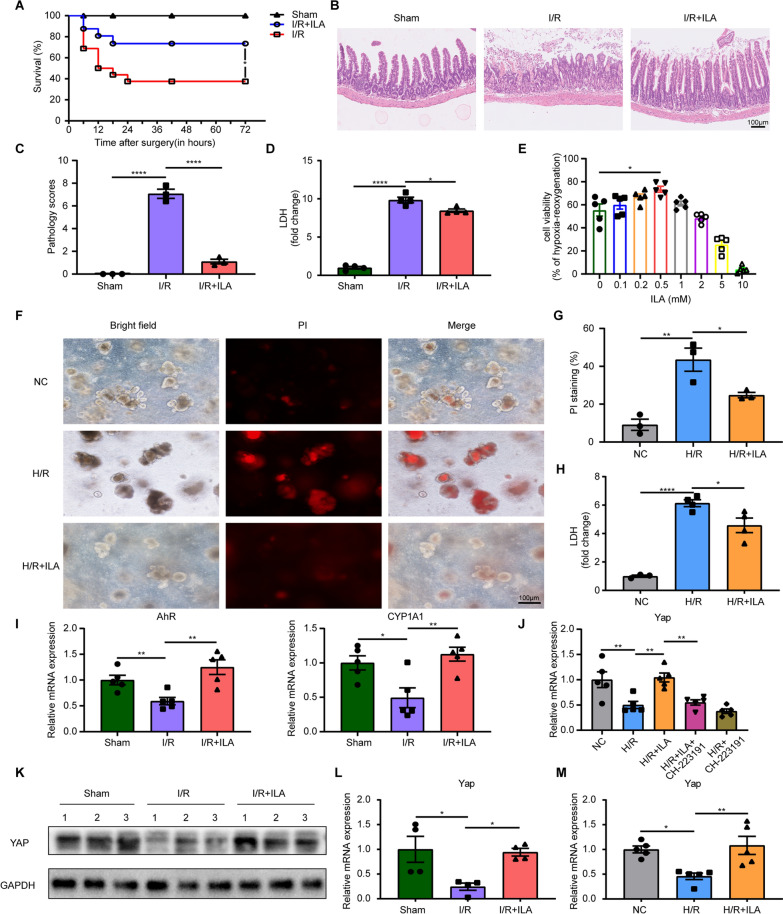
ILA improved the survival rate and inhibited apoptosis of enterocytes under I/R and H/R injury. **A** Survival rate between sham, I/R and I/R + ILA groups (n = 16/group). **B-C** H&E staining (**B**) and quantification (**C**) of the histopathology changes of intestinal tissue sections (n = 3/group). **D** Relative serum LDH levels in mice under intestinal I/R injury (n = 4/group). **E** The effect of different ILA concentration on organoids’ cell viability under H/R injury (n = 5/group). **F** Representative images of morphological changes and PI staining in organoids. **G** Quantification of PI staining (n = 3/group). **H** Relative LDH levels in the organoids culture supernatant (n = 3–4/group). **I** AhR and CYP1A1 mRNA levels in intestinal tissues (n = 5/group). **J** YAP mRNA levels in organoids (n = 5/group). **K** Western blot for the protein expression of YAP in intestinal tissues (3 representative cases in each group). **L** YAP mRNA levels in intestinal tissues (n = 4/group). **M** YAP mRNA levels in organoids (n = 5/group). Results are presented as mean ± SEM. The statistical tests employed included: two-tailed log-rank test in (**A**), two-tailed student’s t-test in **C**–**E**, **G**–**I**, **L** and **M** and one-way ANOVA followed by the Tukey test for multiple comparisons in **J**. * p < 0.05, ** p < 0.01, **** p < 0 .0001

To determine the effect of ILA on intestinal I/R injury, the mice in the ILA group were administrated 20 mg/kg ILA, while the same group received corn oil. The administration of ILA significantly increased the survival rate of I/R models (Fig. 2A). We then investigated the protective role of ILA in murine intestinal I/R injury models to prevent intestinal damage. The intestinal tissue in I/R group showed enterocyte apoptosis, edema and loss of intestinal villus. In contrast, the I/R + ILA group showed reduced ulcers, integrated villus and mild edema (Fig. 2B, C). Moreover, the I/R group exhibited a higher level of LDH in comparison to the control group, while ILA treatment reduced the LDH level (Fig. 2D).

Intestinal organoid damage also occurred after H/R injury. We assessed the cellular ATP content for the organoids cell viability detection. After pretreatment with ILA in concentration ranging from 0.1 to 10 mM for 24 h, the highest cell viability of organoids under H/R damage was observed with 0.5 mM ILA administration compared to the control group (Fig. 2E). We used the effective dosage of ILA for the subsequent studies. Propidium iodide (PI) cannot pass through the membrane of living cells, thus providing a sensitive and robust method for detecting and quantifying intestinal epithelium apoptosis, revealing abundant cell death [31]. Consistent with cell viability results, after H/R, PI staining showed that over 40% of the organoids were disrupted, and exhibited increased apoptosis. Nevertheless, apoptosis was reduced in the H/R + ILA group (Fig. 2F, G). Also, the H/R + ILA group exhibited lower LDH levels, representing decreased enterocyte injury compared to the H/R group (Fig. 2H).

Since the ILA was reported to be an AhR agonist, we further examined the activation of AhR and its target gene CYP1A1. As shown in Fig. 2I, the mRNA level of AhR and CYP1A1 were significantly increased in I/R + ILA group as compare to I/R group. Recently, AhR has been reported to be associated with stemness maintenance and regulation [32]. We then analyzed the mRNA level of YAP in organoids. We found that the up-regulation of YAP in the H/R + ILA group was abolished by AhR-specific inhibitor CH-223191 (Fig. 2J). These results suggested that AhR was involved in ILA-induced activation of YAP. Additionally, the protein expression of YAP was determined by western blot, with three representative samples in each group shown in Fig. 2K. The YAP levels decreased in the I/R group when compared to the control group. Whereas, administration with ILA in the I/R group increased the expression of YAP protein. In addition, the mRNA level of YAP was down-regulated in the I/R group, while up-regulated in the I/R + ILA group (Fig. 2L). Furthermore, the mRNA level of YAP decreased in the H/R group, but increased in the H/R + ILA group in organoids (Fig. 2M). These findings suggest that ILA administration plays a protective role in intestinal I/R injury, improving the expression of YAP protein through AhR.

### Inhibition of YAP reversed the protective role of indole-3-lactic acid in intestinal I/R injury

**Fig. 3:**
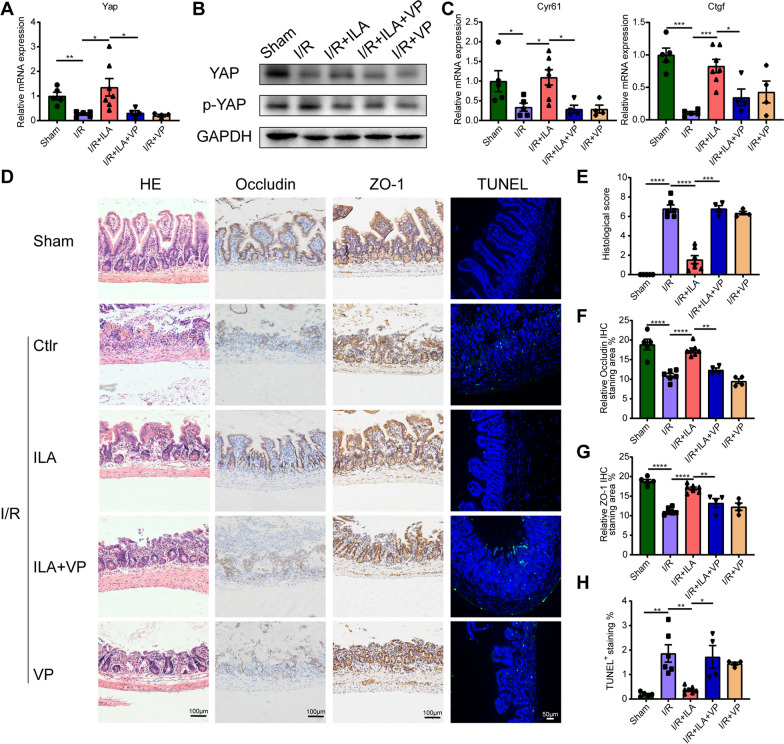
YAP mediated the protective role of ILA in intestinal I/R injury. **A**-**B** Gene expression level (**A**) (n = 4–7/group) and protein expression level (**B**) (Four independent experiments) in intestinal tissues. **C** YAP downstream gene Cyr61, Ctgf expression in the intestinal tissues (n = 4–7/group). **D** H&E staining of the histopathological changes of intestinal tissues, immunohistochemistry staining of intestinal barrier tight junction protein (ZO-1 and Occludin), TUNEL staining of enterocyte apoptosis. **E**-**H** Quantification of pathological scores (**E**), relative levels of Occludin (**F**) and ZO-1 (**G**) proteins and the proportion of TUNEL^+^ cells (**H**) (n = 4–7/group). Results are presented as mean ± SEM. The statistical tests employed included: one-way ANOVA followed by the Tukey test for multiple comparisons in **A**, **C** and **E**–**H**. * p < 0.05, ** p < 0.01, *** p < 0 .001, **** p < 0 .0001

In order to explore whether the effects of ILA on I/R injury depend on the expression of YAP protein, mice were pre-treated with VP, a small molecular YAP inhibitor [33]. We found that ILA treatment significantly increased the mRNA level and protein expression of YAP, decreased the phosphorylation of YAP compared to the I/R group (Fig. 3A, B). In contrast, VP administration significantly decreased the mRNA level and protein expression of YAP compared to the I/R + ILA group (Fig. 3A, B). Likewise, VP administration significantly decreased the expression of YAP downstream target genes Cyr61 and Ctgf, which are involved in cell proliferation, compared to the I/R + ILA group (Fig. 3C). These data suggest that VP can inhibit YAP protein expression. Histopathology revealed that ILA administration significantly alleviated apoptosis of epithelial cells, crypt distortion as well as severe mucosal barrier damage induced by I/R injury, while VP administration reversed these effects (Fig. 3D, E). Immunohistochemistry staining showed that VP administration failed to improve the expression of Occludin and ZO-1 proteins compared to the I/R + ILA group (Fig. 3D, F, G). TUNEL staining showed that administration of VP reversed the protective role of ILA (Fig. 3D, H).

**Fig. 4:**
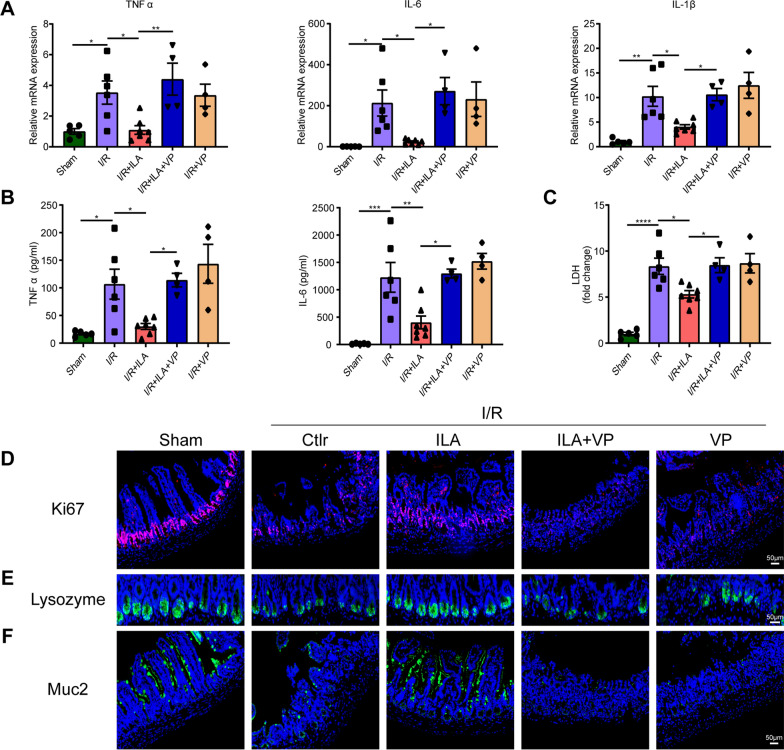
ILA alleviated intestinal inflammation and promoted intestinal regeneration by promoting YAP expression. **A** Gene expression levels of TNF-α, IL-6 and IL-1β in intestinal tissue (n = 4–7/group). **B** Cytokines TNF-α and IL-6 levels in serum (n = 4–7/group). **C** Relative LDH levels in the serum (n = 4–7/group). **D**-**F** Immunofluorescence of Ki67 (**D**), lysozyme (**E**) and Muc2 (**F**) in intestinal tissue sections. Results are presented as mean ± SEM. The statistical tests employed included: one-way ANOVA followed by the Tukey test for multiple comparisons in **A**–**C**. * p < 0.05, ** p < 0.01, *** p < 0.001, **** p < 0.0001

Moreover, reduced expression of YAP protein in the I/R + ILA + VP group resulted in increased local inflammation of intestinal tissue and systemic inflammation, as evidenced by increased mRNA levels of TNFα, IL-1β and IL-6 in intestinal tissue (Fig. 4A), up-regulated cytokines including TNFα and IL-6 in serum (Fig. 4B) compared to the I/R + ILA group. Additionally, ILA administration significantly decreased LDH levels in serum compared to the I/R group, while VP pretreatment significantly increased LDH levels compared to the I/R + ILA group (Fig. 4C). Conclusively, YAP mediates the protective role of ILA in treating intestinal damage under I/R injury.

### Indole-3-lactic acid promoted the self-renewal of ISCs after intestinal I/R injury

The effects of ILA on epithelial cell proliferation were evaluated by staining intestinal tissue sections with Ki67. The expression of Ki67 protein was significantly higher in the I/R + ILA group when compared to the I/R group (Fig. 4D). Additionally, the relative mRNA level of Ki67 was higher in the I/R + ILA group than in the I/R group (Additional file 2: Figure S1A). YAP expression is closely related to stem cell proliferation and differentiation [34], therefore the active proliferating ISCs markers Lgr5 and Ascl2 were detected in this study. We found that the expression of Lgr5 and Ascl2 was higher in ILA pre-treated mice (Additional file 2: Figure S1B, C). These data indicate the role of ILA in promoting the self-renewal of ISCs.

Studies have suggested that Paneth cells play vital roles in stem cell maintenance and renewal by expressing TGF-α, EGF, Wnt3, and notch ligand, DLL4 [35]. Lysozyme, a Paneth cell marker, restores the anti-bacterial activity of Paneth cells and maintains the activity of the stem cell niche. These functions of Paneth cells have effectively alleviated irritable bowel syndrome [36]. In this study, lysozyme expression in I/R mice was lower compared to the sham group. However, the administration of ILA increased lysozyme production in I/R + ILA mice, while the effects were reversed in the I/R + ILA + VP group (Fig. 4E). Interestingly, administration of ILA also led to an increase in Muc2^+^ positive goblet cells, indicating a positive effect of ILA on intestinal secretory functions (Fig. 4F). Taken together, these results suggest that ILA contributes to intestinal epithelial cell proliferation and self-renewal of ISCs, which were reversed by VP administration.

### Indole-3-lactic acid administration reduced apoptosis and promoted the proliferation of organoids under H/R injury

**Fig. 5:**
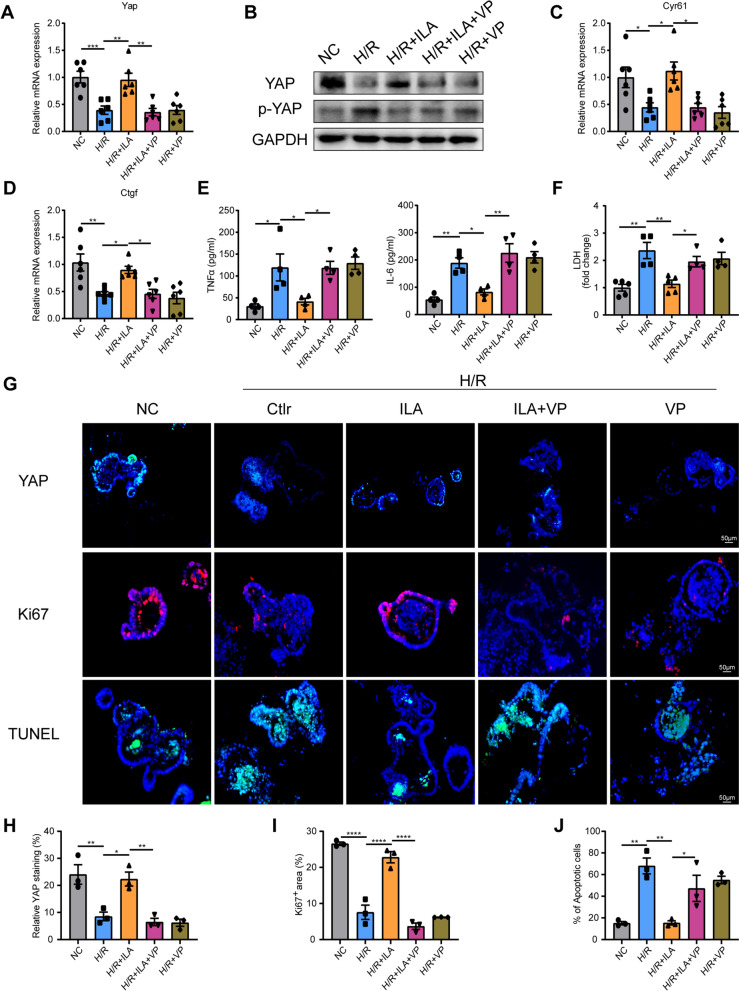
ILA protected intestinal organoids against H/R injury in vitro. **A** YAP mRNA expression in intestinal organoids (n = 6/group). **B** Representative images of protein levels in intestinal organoids (Four independent experiments). **C**-**D** YAP downstream gene Cyr61 (**C**), Ctgf (**D**) expression in intestinal organoids (n = 6/group). **E** ELISA analysis of the cytokine TNF-α and IL-6 in the supernatant medium (n = 4/group). **F** Relative LDH levels in the supernatant medium (n = 4–5/group). **G** Immunofluorescence of YAP and Ki67 in intestinal organoids frozen sections, TUNEL staining in intestinal organoids frozen sections. **H**-**J** Quantification of YAP protein expression (**H**), Ki67^+^ cells (**I**) and TUNEL^+^ cells (**J**) in organoids (n = 3/group). Results are presented as mean ± SEM. The statistical tests employed included: one-way ANOVA followed by the Tukey test for multiple comparisons in **A**, **C**, **D**–**F** and **H**–**J**. * p < 0.05, ** p < 0.01, *** p < 0 .001, **** p < 0 .0001

We conducted futher investigation into the modulatory effect of ILA on intestinal organoids. Similar to our in vivo studies, the H/R + ILA group exhibited significantly increased YAP mRNA and protein levels compared to the H/R group and H/R + ILA + VP group (Fig. 5A, B, G, H). Moreover, ILA elevated the mRNA levels of YAP target genes, Cyr61 and Ctgf under H/R conditions, which was eliminated by pretreatment with VP (Fig. 5C, D). We also noted a reduction in cytokines such as TNFα and IL-6 in the organoids supernatant after ILA administration under H/R injury compared to the H/R group and H/R + ILA + VP group (Fig. 5E). Furthermore, ILA significantly reduced LDH levels in the supernatant of organoids under H/R injury as compared to the H/R group and H/R + ILA + VP group (Fig. 5F). We also confirmed increased proliferative activities of enterocytes in the H/R + ILA group as compared to the H/R group and H/R + ILA + VP group, evidenced by increased expression of Ki67^+^ in organoids (Fig. 5G, I). These findings suggest that ILA enhances the activation of YAP expression, thereby stimulating the proliferation of intestinal epithelial cells and progenitor cells, which are the primary ways to replenish damaged cells. Additionally, ILA administration reduced the organoids apoptosis than in the H/R group and H/R + ILA + VP group as presented in TUNEL immunostaining (Fig. 5G, J). Conclusively, ILA reduced the release of inflammatory cytokines after organoid damage initiated by H/R and restored the proliferation of enterocytes, which can be abolished by VP treatment. These data suggest that YAP is necessary for ILA in promoting proliferation and alleviating apoptosis under H/R stress.

### Nrf2 is necessary for indole-3-lactic acid in alleviating oxidative stress under intestinal I/R

**Fig. 6:**
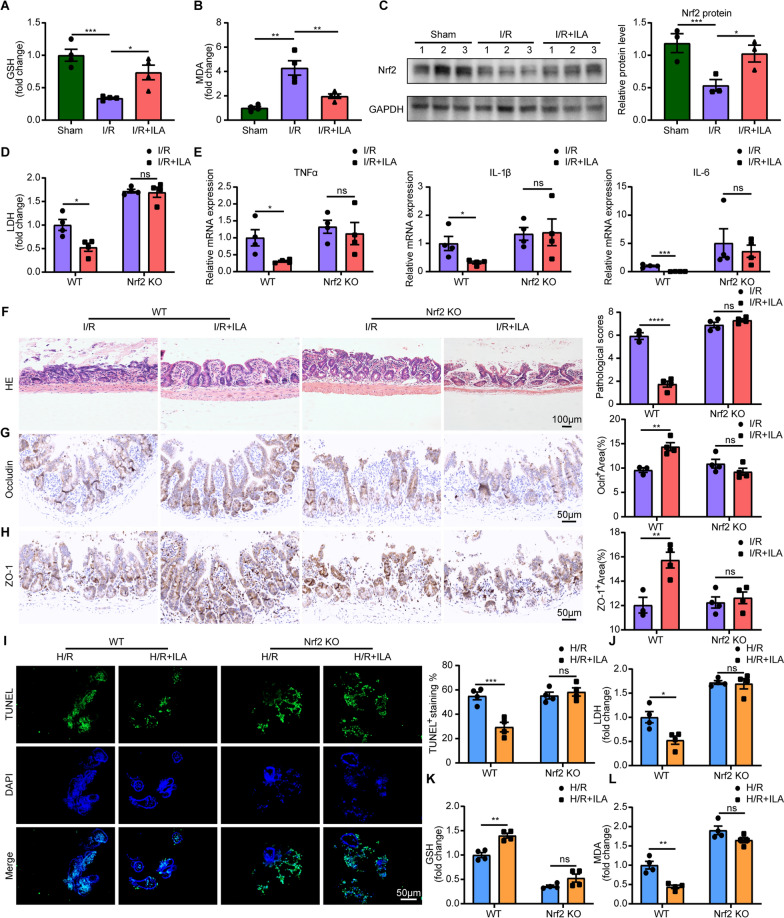
ILA reduced excessive inflammation and oxidative stress in enterocytes both in vivo and in vitro. **A**-**B** Relative GSH level (**A**) and MDA level (**B**) in the serum (n = 4/group). **C** Representative images and quantification of the protein level of Nrf2 in intestinal tissue (3 representative cases in each group). **D** Relative LDH level in the serum (n = 4/group). **E** Relative gene expression of TNFα, IL-1β and IL-6 in intestinal tissue (n = 4/group). **F**-**H** Representative images and quantification of HE staining (**F**), immunohistochemical staining of Occludin (**G**) and ZO-1 (**H**) protein expression in the intestine tissues (n = 3–4/group). **I** Representative images and quantification of TUNEL^+^ cells in intestinal organoids frozen sections. **J**-**L** Relative LDH level (**J**), GSH level (**K**) and MDA level (**L**) in the culture supernatants of organoids (n = 4/group). Results are presented as mean ± SEM. The statistical tests employed included: two-tailed student’s t-test in **A**–**C**, two-way ANOVA followed by the Tukey test for multiple comparisons in **D**–**L**. * p < 0.05, ** p < 0.01, *** p < 0.001, **** p < 0.0001

Oxidative stress often causes high cytotoxicity leading to organ I/R damage [37, 38]. We further investigated the role of ILA in maintaining redox balance. GSH and MDA are reported to be biomarkers of oxidative stress [39]. The depletion of GSH and generation of MDA are associated with oxidant damage. Our findings indicate that ILA significantly increased the GSH level compared with the I/R group (Fig. 6A). While the MDA level was increased in the I/R group, administration of ILA decreased it (Fig. 6B), suggesting the antioxidant role of ILA in intestinal I/R injury. Nrf2 plays an important role in antioxidant and anti-inflammation in I/R injury [40]. Intestinal Nrf2 protein levels increased in the I/R + ILA group but not in the I/R group (Fig. 6C). However, both the I/R and I/R + ILA groups of Nrf2 deficient mice showed similarly high levels of serum LDH (Fig. 6D) and pro-inflammatory cytokine profiles (Fig. 6E). Moreover, there was no difference between the I/R and I/R + ILA groups of Nrf2 deficient mice in terms of intestinal tissue pathological damage, and expression of tight junction proteins ZO-1 and Occludin (Fig. 6F–H). These results suggest that ILA treatment failed to protect Nrf2 deficient mice from intestinal I/R injury.

Consistent with our in vivo studies, both intestinal organoids in the H/R group and H/R + ILA group from Nrf2-deficient mice exhibited obvious apoptosis in epithelial cells (Fig. 6I). Additionally, both intestinal organoids supernatant in the H/R group and the H/R + ILA group from Nrf2-deficient mice showed high levels of LDH (Fig. 6J). This suggests that ILA failed to protect the Nrf2-deficient organoids from damage under H/R stress. Moreover, both GSH (Fig. 6K) and MDA (Fig. 6L) levels showed no statistical difference between organoids supernatant in the H/R group and the H/R + ILA group from Nrf2-deficient mice. These data indicate that ILA failed to alleviate the oxidative stress of Nrf2-deficient organoids from H/R stress. Taken together, these data suggested that Nrf2 is necessary for ILA to exert its protective effects against inflammation and oxidative stress under I/R injury.

## Discussion

In our current study, we report for the first time the correlation of *L. murinus* derived tryptophan metabolites ILA, IAA, and IAld with intestinal function in patients undergoing CPB surgery. Of the three metabolites, ILA showed the most promising results in protecting against intestinal I/R injury. We also discovered that ILA promoted the self-renewal of ISCs and protected epithelial cells from inflammation and oxidative stress by regulating YAP and Nrf2, both in vivo and in vitro. Therefore, our data provides a prospective therapeutic approach and promising candidate target for treating intestinal I/R injury.

Tryptophan metabolites derived from the catabolism of anaerobic bacteria have been well described [15]. These tryptophan metabolites are utilized as intercellular signal molecules [41]. ILA is reported to modulate immune responses and enhance intestinal homeostasis in intestinal inflammation [16, 42]. Recent research has shown that lowered fecal IAA levels may be associated with alcoholic hepatitis in patients [43]. Additionally, 3-IAld treated mice have been reported to have a positive role in protecting against immune checkpoint inhibitors-induced colitis [19]. Taken together, these studies suggest the potential protective effect of ILA, IAA and IAld in intestinal I/R injury. Interestingly, we observed that ILA levels decreased after intestinal I/R injury, whereas IAA and IAld levels remained unchanged. Intestinal FABP is a protein produced by small intestinal enterocytes and released into the serum when intestinal epithelial cells are damaged [44]. Similarly, D-lactic acid, a metabolite of the intestinal flora, is released into the serum when the intestinal barrier function is impaired [44]. Therefore, the measurement of I-FABP and D-lactic acid levels in serum can be used as the biomarker of intestinal damage. By comparing the correlation of fecal content of three *L. murinus*-derived tryptophan metabolites with the LIFE score and serum biomarkers of intestinal injury in patients undergoing CPB surgery, we found that reduced ILA levels significantly correlate with higher LIFE scores, serum I-FABP and D-lactic acid levels. These findings suggest that high abundance of ILA is associated with better prognosis of intestinal function. However, we did not observe a significant correlation between fecal contents of IAA and IAld in the feces of patients undergoing CPB surgery with the score of postoperative gastrointestinal dysfunction or serum biomarkers of intestinal permeability.

Intestinal I/R injury involves two stages: hypoxic cellular stress caused by ischemia and inflammation-associated reperfusion injury. The release of endogenous ROS causes tissue damage, initiates circulatory disturbance as well as a cascade of inflammatory reactions, resulting in death of enterocytes [1–3]. Enterocytes undergo necrosis, apoptosis, and DNA degradation, which ultimately result in the loss of intestinal villi [29]. Growing evidence has suggested that intestinal flora and its metabolites play a positive role in protecting intestinal barrier function and maintaining intestinal homeostasis [25, 45, 46]. Consistent with our previous finding that *L. murinus* contribute to mitigate intestinal epithelial permeability and prevent bacterial translocation in I/R injury models [13], our study found that the administration of *L. murinus*-derived tryptophan metabolites ILA significantly improved the epithelial barrier function through maintaining the tight junction proteins and mucins protein in mice undergoing intestinal I/R injury. Previous studies report that ILA has antioxidant and immunomodulation abilities [14, 47]. On a similar note, the current study found that ILA treatment protects against intestinal injury from inflammation and expands regenerative programs in I/R injury models (in vivo). These conclusions were also verified in the H/R stress intestinal organoids (in vitro). Mechanistically, pretreatment of mice and organoids with ILA increased the expression of YAP.

YAP has been reported to protect cardiomyocytes and neurons from I/R injury by enhancing antioxidant gene expression and activating transcriptional programs [48, 49]. Our study explored, for the first time, the relationship between YAP and intestinal I/R injury, and found that YAP expression decreased in the I/R group as compared to the sham group, indicating that YAP is essential for the maintenance of intestinal homeostasis under cellular stress. Previous studies have shown that increased expression levels of key YAP target genes such as Ctgf and Cyr61 prevent apoptosis and promote chemoresistance [50]. To further evaluate the underlying mechanism of ILA in treating intestinal I/R injury, we proved that treatment with ILA promoted downstream regenerative (Ctgf, Cyr61) gene profiles of HIPPO signaling, diminished LDH production, reduced enterocyte exudative necrosis/apoptosis and reduced pro-inflammatory cytokine expression in I/R mice. Compared to the single intestinal epithelial cell line, intestine organoids have multiple functional cell types, which can provide more specific and accurate disease model for mechanisms researches. In our study, we employed H/R stressed organoid cultures to simulate the intestinal I/R injury in vitro. We found that administration of ILA promoted the expression of YAP in the nucleus of multiple cell types. While the nuclear localization of yap can promote the proliferation and maintenance of ISCs, which is of great importance when a large number of villus and crypts are lost during intestinal I/R injury. Pretreatment with ILA increased YAP expression and eliminated inflammation injury. However, administering the YAP inhibitor reversed the protective effects of ILA on treating H/R-associated organoid damage and eliminated the regenerative function of ILA administration in vitro. Therefore, YAP is necessary for the protective effects of ILA in treating intestinal I/R injury.

The continuous proliferation and differentiation of ISCs form the basis of intestinal epithelial cell renewal and regeneration [51]. Under elevated levels of pro-inflammatory cytokines such as TNF-α, IL-6 and IL-1β, ISCs are damaged and inactivated, resulting in delaying tissue recovery. Mutant YAP has been observed to decrease cell proliferation and increase susceptibility to colitis [52]. In our mouse intestinal I/R model, ILA increases YAP expression thereby preventing intracellular damage by regulating cell proliferation and promoting ISCs differentiation into various cell types. YAP is the core regulator of cell growth and cell death [53, 54]. Up-regulated YAP in stem cells prolongs their activation, leading to function reconstitution [53, 54]. Here, YAP activation promotes ISCs differentiation into secretory cells, such as goblet cells and Paneth cells, further eliminating innate inflammatory responses both in vivo and in vitro.

Our research demonstrated that ILA protected mice undergoing intestinal I/R injury from oxidative stress and excessive inflammation. The transcription factor Nrf2 regulates the resistance of cells to oxidative stress and is considered to be the “master switch” of redox homeostasis in cells [55]. Nevertheless, the relationship between ILA and Nrf2 in intestinal I/R injury has not yet been established. Our study proved that the administration of ILA promoted the expression of Nrf2 protein in the intestinal tissue of mice under I/R stress. We further confirmed that ILA failed to protect against intestinal injury from I/R damage in Nrf2-deficient mice, as evidenced by elevated levels of LDH and increased inflammatory cytokines. Interestingly, in our study, intestinal organoids cultured from Nrf2-deficient mice were shown to be more susceptible to H/R-mediated damage. Literature suggests that Nrf2 exerts an important role in various leukocytes [56], while the lack of immune cells in the organoids model indicates that the significant anti-oxidant effect of Nrf2 in epithelial cells plays a decisive role under H/R stress. These findings elucidate the significance of Nrf2 in promoting the anti-oxidative effects against I/R and H/R damage, which helps maintain balance in the bowel microenvironment. Collectively, our results confirm that ILA can inhibit intestinal I/R injuries as well as organoid H/R injuries by positively regulating of YAP and Nrf2.

Here, we demonstrate that decreased levels of ILA in preoperative feces of patients are associated with poor gastrointestinal functions prognosis after CPB surgery. However, as is common in human studies, our results are only relevant and the impact of supplementation with ILA on gastrointestinal function in patients after CPB surgery remains to be further studied. In addition, our focus is on promoting proliferation and anti-oxidative function of ILA. While previous studies have shown that ILA can also affect immune function as an AhR agonist, the effect of ILA on cellular immunity requires further research.

## Conclusions

In summary, our findings indicate that the fecal content of microbiota-derived tryptophan metabolites ILA is associated with intestinal function and dietary supplementation with ILA could represent a potential treatment for future translational applications in intestinal I/R injury. Our results show that administration of ILA reduces intestinal tissue damage and inflammation, promotes the proliferation and differentiation of intestinal ISCs through the regulation of YAP and alleviates oxidative stress via the regulation of Nrf2 both in vivo and in vitro. This study can help reveal a new therapeutic strategy for clinical practice and elucidate a novel mechanism of intestinal I/R injury. Future efforts to protect intestinal I/R injury by supplementing the end products of microbiota may serve a high clinical efficacy.

## Supplementary Information

**Aditional file 1: Table S1.** Primer sequences.

**Additional file 2: Figure S1.** ILA promoted ISCs proliferation. (A) Ki67 mRNA levels in intestinal tissues (n = 4–5/group). (B) Lgr5 mRNA levels in intestinal tissues (n = 4–5/group). (C) Ascl2 mRNA levels in intestinal tissues (n = 4–5/group). Results are presented as mean ± SEM. * p < 0.05, ** p < 0.01.

## Data Availability

All data supporting the conclusions of this research are included in this paper, any further supporting data can be queried with the corresponding author.

## Abbreviations

*L. murinus*: *Lactobacillus murinus*
I/R: Ischemia/reperfusion
H/R: Hypoxia/reoxygenation
ILA: Indole-3-lactic acid
YAP: Yes Associated Protein
Nrf2: Nuclear Factor erythroid 2-Related Factor 2
VP: Verteporfin
CPB: Cardiopulmonary bypass
MODS: Multiple organ dysfunction syndrome
ROS: Reactive oxygen species
IAA: Indole-3-acetic acid
IAld: Indole-3-aldehyde
AhR: Aryl hydrocarbon receptor
LIFE: Lausanne Intestinal Failure Estimation
LDH: Lactate dehydrogenase
