# Supplementary material for: Microbiota-derived tryptophan metabolites indole-3-lactic acid is associated with intestinal ischemia/reperfusion injury via positive regulation of YAP and Nrf2
Source: J Transl Med. 2023 Apr 18;21:264. doi: 10.1186/s12967-023-04109-3 (PMC10111656; doi:10.1186/s12967-023-04109-3)
Supplement: Supplementary file 1 — Aditional file 1: Table S1. Primer sequences. [file 12967_2023_4109_MOESM1_ESM.docx]

**Supplementary Table 1 Primer Sequences**

| Gene | Forward primer (5'-3') | Reverse primer (5'-3') |
| --- | --- | --- |
| 18S | CGATCCGAGGGCCTCACTA | AGTCCCTGCCCTTTGTACACA |
| YAP1 | CCTGATGGATGGGAGCAAGC | ACTCTGAGTGATCCTCTGGTTC |
| Ctgf | AGACACATTTGGCCCTGACC | TCTTAGAACAGGCGCTCCAC |
| Cyr61 | GCTGGAATGCAATTTCGGCG | TTGGAAGCTCTCCCCGTTCT |
| IL-1β | TGTGAAATGCCACCTTTTGA | GGTCAAAGGTTTGGAAGCAG |
| IL-6 | TGATGCACTTGCAGAAAACA | ACCAGAGGAAATTTTCAATAGGC |
| TNFα | CCACCACGCTCTTCTGTCTAC | AGGGTCTGGGCCATAGAACT |
| Ascl2 | AAGCACACCTTGACTGGTACG | AAGTGGACGTTTGCACCTTCA |
| Ki67 | ATCATTGACCGCTCCTTTAGGT | GCTCGCCTTGATGGTTCCT |
| Lgr5  AhR  CYP1A1 | CCTACTCGAAGACTTACCCAGT  CAAATCCTTCTAAGCGACACAG  CAATGAGTTTGGGGAGGTTACTG | GCATTGGGGTGAATGATAGCA  TGACGCTGAGCCTAAGAACA  CCCTTCTCAAATGTCCTGTAGTG |
