# Supplementary figures and images for: Microbiota-derived tryptophan metabolites indole-3-lactic acid is associated with intestinal ischemia/reperfusion injury via positive regulation of YAP and Nrf2
Source: J Transl Med. 2023 Apr 18;21:264. doi: 10.1186/s12967-023-04109-3 (PMC10111656; doi:10.1186/s12967-023-04109-3)

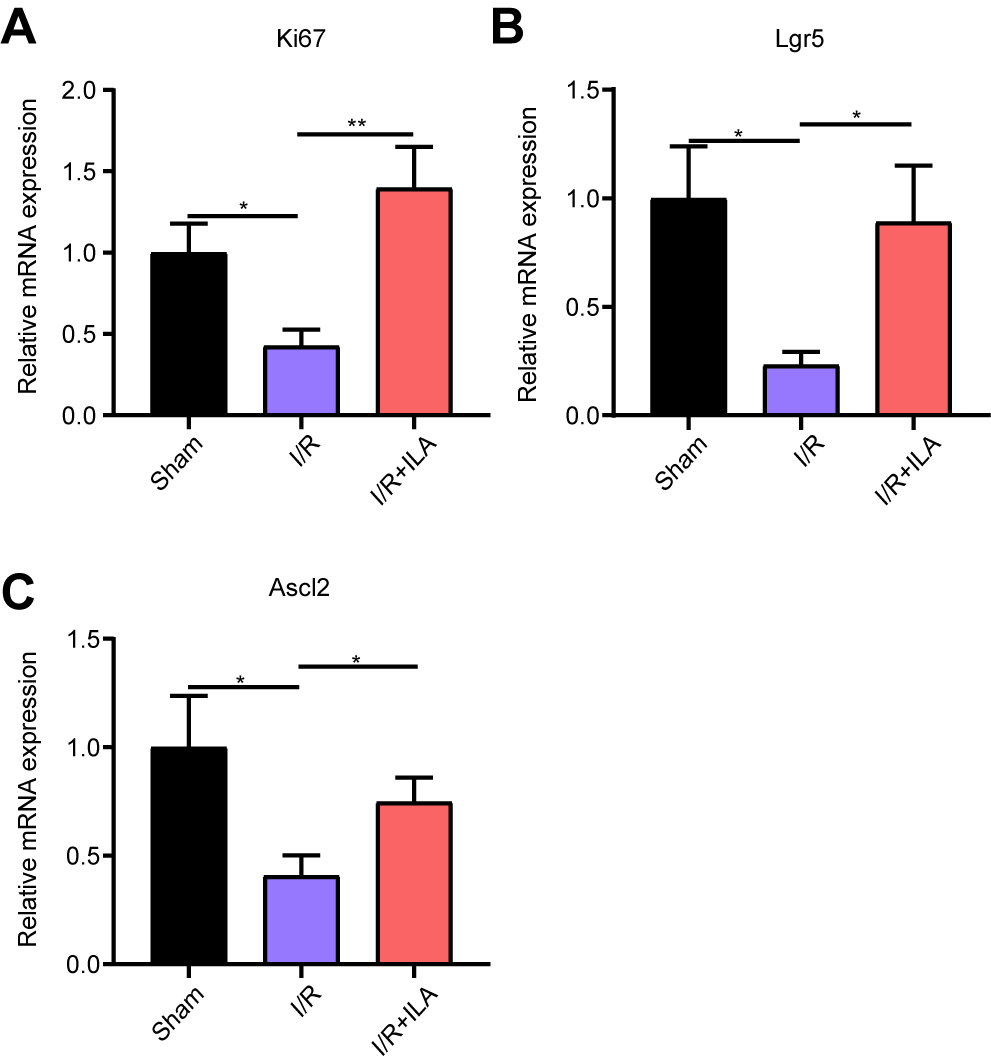

Supplement: Supplementary file 2 — Additional file 2: Figure S1. ILA promoted ISCs proliferation. (A) Ki67 mRNA levels in intestinal tissues (n = 4–5/group). (B) Lgr5 mRNA levels in intestinal tissues (n = 4–5/group). (C) Ascl2 mRNA levels in intestinal tissues (n = 4–5/group). Results are presented as mean ± SEM. * p < 0.05, ** p < 0.01. [file 12967_2023_4109_MOESM2_ESM.tif]
